# Does exposure to social media content influence attitudes towards, and engagement in, road rule violations? A systematic review

**DOI:** 10.1371/journal.pone.0275335

**Published:** 2022-09-28

**Authors:** Kayla B. Stefanidis, Ben Davey, Verity Truelove, Carla Schiemer, James Freeman

**Affiliations:** Road Safety Research Collaboration, University of the Sunshine Coast, Sippy Downs, Queensland, Australia; Zhejiang University, CHINA

## Abstract

**Background/objectives:**

With the increasing popularity and saliency of social media, there is a pressing need to identify whether exposure to such content can affect road rule compliance, especially given that social media has been found to influence other risky behaviours. This systematic review (conducted in accordance with the PRISMA guidelines) summarised existing evidence concerning: (a) the nature of driving-related content on social media and (b) whether such content can influence attitudes and subsequent driving behaviour.

**Methods:**

Peer-reviewed articles written in English, that explored social media content in relation to road safety or driving behaviours (e.g., speeding, tailgating, distraction, impaired driving, and seatbelt use), were eligible for review. Searches were conducted via SCOPUS, PUBMED, ProQuest and TRID in June 2021.

**Results/discussion:**

A total of 8 studies met the requirements for this study, resulting in three key findings. First, it was found that very few studies have explored the type and extent of driving-related content on social media, and the small collection of existing research has focused solely on YouTube and Twitter. Second, whilst the nature of driving-related content on social media varies substantially across studies, a body of content exists that promotes or encourages risky driving behaviour or road rule violations. Third, and despite the array of available online content, there is a paucity of research illuminating the impact of social media messages on attitudes towards, and behaviours linked to road safety. This review highlights the need for research to keep pace with the rapidly changing nature of social media (not least the impacts upon human behaviour) and outlines pathways to increase current scientific understanding.

## Introduction

Social media has become the dominant medium for information exchange, communication, and self-expression in the modern world [1, 2]. The number of users on social media (e.g., Facebook, Instagram, YouTube and Twitter) has surpassed 400 million, and is expected to increase exponentially in the coming years [3]. Given the rapid expansion and increasing connectedness offered by social media, there is a growing need to explore and understand the subsequent effect of exposure upon attitudes and behaviours, particularly whether such exposure can promote adverse effects. For example, there is preliminary evidence accumulating to suggest that exposure to content depicting or encouraging risky behaviours (e.g., alcohol use, substance use, suicidality or disordered eating), can increase one’s likelihood of engaging in the behaviour [4–7]. However, one question that remains to be answered is whether social media can affect (and to what extent) attitudes towards, and engagement in, road rule violations. This is a major shortcoming, given: (a) that billions rely on automobiles for transport, (b) that social media has been shown to influence a range of negative behaviours and (c) considering the ongoing high road toll. In regard to the latter, the problem of crashes is well documented and accounts for 1.3 million deaths and 20–50 million injuries each year, with at least two thirds of these resulting from human error and illegal driving behaviours, including speeding, impaired driving and driver distraction [8]. Despite increased efforts to reduce engagement in such behaviours, road crash fatality rates have been increasing, as opposed to decreasing, in various areas worldwide [9]. From a human learning theory perspective, regular exposure to content online that encourages or promotes risky driving behaviour has the potential to normalise or encourage the behaviour (via modelling mechanisms), which can in turn increase the likelihood of individuals engaging in the behaviour themselves [10–12]. It may be suggested that drivers’ increased exposure to road rule violations via social media could be contributing to drivers’ engagement in deliberate aberrant behaviours.

However, it should be noted that not all content on social media encourages risky driving behaviour and in fact, some content may promote safe driving behaviours. For example, recent studies have revealed social media interventions can effectively reduce smoking or drinking behaviour [13, 14]. However, the needed frequency of exposure and required level of saliency of imagery/messaging remains almost completely unknown. Given this, a comprehensive examination of the relationship between social media exposure to different types of driving stimuli/imagery and subsequent driving behaviour is clearly needed in order to (a) illuminate the extent (if any) of the risk and (b) form a foundation for the development of strategies to mitigate such risk. Accordingly, the current study aims to implement a systematic review (in accordance with the PRISMA guidelines) to summarise existing evidence concerning: (a) the nature of driving-related content on social media and (b) whether such content can influence attitudes and subsequent driving behaviour.

## Method

This review was conducted in accordance with the Preferred Reporting Items for Systematic Reviews and Meta-Analyses (PRISMA) guidelines [15]. Since no participants were involved in this research, ethics approval was not required. Given the small number of studies identified, a protocol document was not prepared. Further, the review was not registered.

### Search strategy

Literature searches and preliminary screening were conducted by one author (BD) in June 2021. Title/abstract screening and study selection was performed by two authors (BD and KS), with KS and VT both overseeing the selection process. The following databases were utilised: SCOPUS, PUBMED, ProQuest and TRID (see S1 File). Searches were limited to peer-reviewed articles and the English language. Review papers were excluded. Citations were first exported into endnote, where duplicates were deleted. Remaining titles and/or abstracts were subsequently imported into Rayyan [16] for screening.

Studies were eligible for review if the title/abstract included a minimum of one keyword from each of the following categories:

1. Social media: *social media; YouTube; Snapchat; Facebook; Instagram; Twitter; TikTok; Reddit; WhatsApp; Waze; Maps; Navigation Applications*
2. Driving: *Traffic*, *road*, drive*, *driving*
3. Behaviour: *Following distance*, *headway*, tailgat*, *dangerous*, *unsafe*, *distraction*, *texting*, *cellphone*, *phone*, *violation*, offend*, hoon*, *rules*, *drug*, *drink*, *impaired*, *seatbelt*, *speeding*, *speed limit*

Note that navigation applications were included under the category of social media as many applications (e.g., Waze or Google Maps) allow users to share the locations of enforcement cameras or police.

## Data synthesis

Due to the nature of the data, quantitative analyses could not be performed via meta-analysis. As such, the data were analysed qualitatively instead. Key findings and themes within each article pertaining to social media and driving are discussed.

## Results

### Study selection

**Fig 1:**
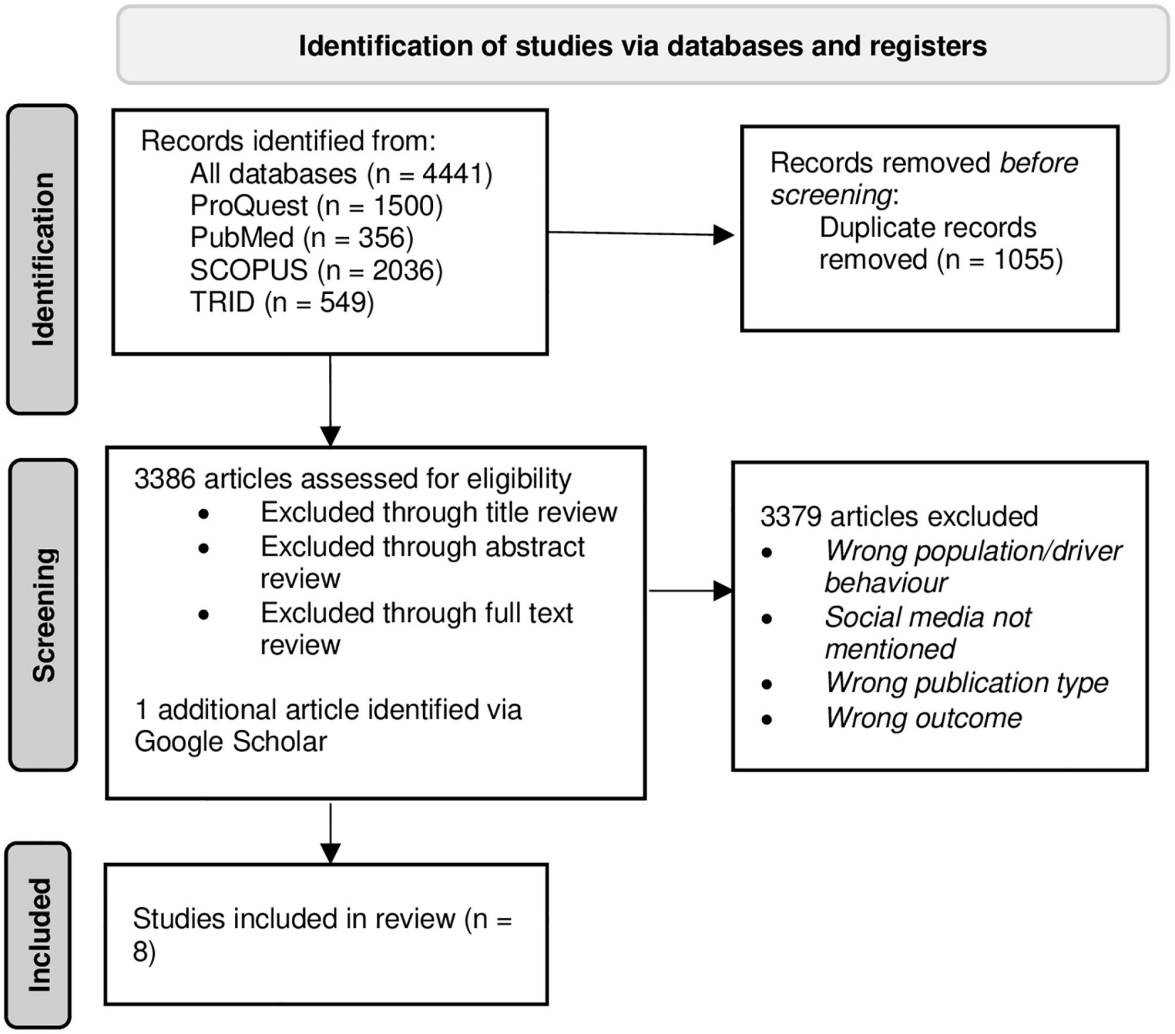
Search strategy and PRISMA flow diagram.

A total of 4441 articles were identified via ProQuest (*n* = 1500), PubMed (*n* = 356), SCOPUS (*n* = 2036), and TRID (*n =* 549), respectively. Of these, 1055 were identified as duplicates. Of the remaining 3386 citations, 3379 articles were not relevant (e.g., did not pertain to road rule compliance or social media) or did not focus on a driving behaviour of interest (e.g., examined driver sleepiness), resulting in 7 articles eligible for review (see Fig 1 for screening process and flow diagram). Note that driver sleepiness was not included in the review, as we were interested specifically in engagement in deliberate aberrant behaviours. Two relevant review articles were screened for additional references, although no additional articles were identified. In addition, one article was identified via Google Scholar in August 2021. Data extraction was performed by two independent authors (KS and CS), and reviewed by VT.

### Study characteristics

Overall, a total of three studies examined distracted driving, whilst the remaining pertained to speeding (*n =* 1), road rage/aggressive driving (*n =* 1) and risky driving behaviours (e.g., stunts or wheelies, *n* = 2). In addition, one study examined attitudes towards road safety as well as high-risk groups and behaviours. In terms of social media, four studies focused on YouTube content, three focused on Twitter, and the final article examined general social media blogs/chat rooms or webpages. Seven studies analysed the nature of the content on the respective social media platform (*n =* 7), whilst only one study explored the potential for YouTube content to influence behaviour using focus groups. Study characteristics and their respective findings can be found in Table 1.

**Table 1:** Study characteristics & key findings.

Article	Population & methodology	Social media platform	Driver behaviour	Key findings of interest
Basch et al., 2019	Content analysis of distracted driving videos on YouTube (included 100 most viewed videos) uploaded between 2007 and 2016	YouTube (comparison of internet-based and television-based videos, as well as professional and consumer uploaded videos)	Distracted driving (phone use)	Combined number of views across the YouTube videos studied = over 35 million. The sample included expert generated videos (n = 1), general consumer uploaded videos (n = 19), television-based videos (videos that were made for television but were also uploaded on YouTube; n = 42) and internet-based videos (with no ties to a television network; n = 38). Phone use while driving was mentioned 13 times more often on television-based videos than consumer-based videos. Television-based videos stated that the behaviour was illegal more often than consumer-based videos (odds ratio = 13). Phone use while driving was mentioned 6.6 times more often on internet-based videos than consumer-based videos. Internet-based videos stated that the behaviour was illegal more often than consumer-based videos (odds ratio = 18). A total of 92/100 videos mentioned phone use while driving. A total of 87/100 videos mentioned texting while driving. A total of 47/100 videos mentioned using social media while driving. Only 1/100 video promoted intentional distractions while driving.
Gjorgjievski et al., 2020	Content analysis of distracted driving videos on YouTube (included videos >3000 views. A total of 788 videos were eligible for inclusion, shared from 2006 to 2018).	YouTube	Distracted driving	Combined number of views across the YouTube videos studied = 223 million. Key content included texting while driving (64.6% n = 509) and talking on the phone while driving (including hand-held and hands-free operations, 24.5% n = 193/788). 81.6% of the videos were serious and did not contain humorous material. A total of 742 (94.2%) of the videos mentioned crashes or death as a consequence of distracted driving, while 166 (21.1%) mentioned injuries as a consequence of the behaviour. 34.5% of the videos fell under the category of amateur. 37.3% of the videos fell under the category of public service announcements. Only 27 (3.4%) of videos reported data from a peer-reviewed research paper/s.
Mooren et al., 2014	Content analysis of community discussions online regarding speed enforcement in Australia Google search using the following search terms: “Australia nanny state speed enforcement” and “Australia road safety speed enforcement”	Blog comments, social media/chat rooms, internet sites, mass media, social commentary articles	Speeding, specifically anti-speed enforcement	The first search (nanny state) generated 3 million articles and were typically written by political or community members/groups and journalists. The second search (Australia road safety speed enforcement) generated 1.36 million articles and were typically written by researchers or the government. In one article, it was found that 77 per cent of readers (n = 2640) had agreed that Australia was a nanny state. Another blog post mentions that “Australian governments treat people like naughty little children”. Some articles contained disagreements or conflicting views e.g., “State road rules are here to protect lives. And they are working. Sorry to disagree with you” Today Tonight and A Current Affair both shared anti-speed enforcement stories (e.g., “Underhanded Speed Cameras” and a story debunking the effectiveness of speed cameras, respectively) during prime time viewing.
Qian et al., 2019	Content analysis of 9,945 tweets concerning phone use while driving/distracted driving, using the following hashtags: #distracteddriving #textanddrive #techinganddriving	Twitter	Distracted driving	Story regarding the increase in penalties for using a mobile phone while driving was retweeted 98 times. A real-life story where a driver was initially caught for distraction, but also charged for drug driving and having no licence by Police, was the most frequently retweeted story during the study. The word driver was strongly associated with the word charges (.48), as well as cannabis, laid and worse. The word ‘phone’ was the most frequently related word to ‘distracted driving’ in the association analysis. A technology company aiming to target distracted driving was the “most active user”. A regional Police service was the “most visible user”. Media organisations were more popular than individual users.
Seeley et al., 2019	Content analysis of 65 YouTube videos portraying risky driving activities found using the search terms: "street racing" (n = 25), "stunt driving" (n = 21) and "ghost riding" (n = 18).	YouTube	Risky driving	Stunt driving videos were most popular overall (i.e., most likes, subscribers, and videos per channel). Predominantly male and younger drivers were portrayed in all videos, apart from three ghost riding videos where female drivers appeared. Females received a higher mean “net like” compared to males. The nature of the comments linked to the street racing videos were mixed. Overall, 31.3% expressed positive attitudes towards the behaviour/s (e.g., speeding, weaving through traffic, drifting), whilst 31.3% expressed negative attitudes. 30% of ghost riding video comments expressed positive attitudes towards the behaviour (i.e., the behaviour was “good” or “exciting”) The majority of the comments linked to stunt driving videos expressed positive attitudes towards the behaviour/s (e.g., drifting, wheelies) (85.7%). A large proportion of the street racing videos were filmed on urban (76%) or rural roads (48%), whereas a large number of ghost riding videos were filmed on urban roads (66.7%) The consequences of risky driving behaviours were rarely mentioned in the videos (n = 44/65) Only 7 videos included the Police Ghost riding videos were “copycat videos where young men attempted to mimic the original ghost ride music video with their vehicles” (pg. 291).
Stephens et al., 2016	Content analysis of 80,923 tweets posted by an individual whilst driving (data collection period of 13 months). Eligibility criteria included: “contained a clear description of driving related events, were from a driver’s perspective, posted while driving, or in a tense that suggested they were, and related to triggers for or reported reactions to driver anger”	Twitter	Road rage and aggressive driving, posting on social media while driving	Posts uploaded while driving included: text messages, photos of other drivers or their vehicle, or short videos Tweets fell under the following categories: judgements over inappropriate behaviours, perceived hostility, general complaints, traffic conditions and near misses A total of 20972 tweets involved complaints/criticisms towards a drivers’ behaviour/s A total of 11529 tweets included complaints related to the speed of others. Of these, 36% pertained to the slow speed of other drivers. Some tweets stated that drivers were driving too slow under certain conditions e.g., in a certain lane (16%) or weather condition (13%). Only 1% of tweets included complaints for speeding. A total of 4808 tweets included negative comments or complaints pertaining to the driving capacity/skills of others (e.g., learn to drive, drivers can’t drive)
Sujon & Dai, 2021	Sentiment analysis and topic modelling using Twitter (data collection period of 4 years 2015–2019), limited to Washington State	Twitter	Attitudes/beliefs towards road safety and high-risk behaviours/high-risk groups	A total of 5.5 million tweets pertained to the importance of road safety. Of these, 55% of individuals highlighted that traffic safety is important to them, whilst 15% had a neutral stance and 30% thought traffic safety was not important to them. Positive attitudes towards road safety have generally increased over time. However, negative attitudes towards road safety have also increased A total of 10,827 tweets pertained to the topic of preventing fatal crashes/injuries. Of these, 40% expressed negative attitudes preventing such consequences A total of 23,997 tweets pertained to attitudes towards police enforcement of road rule violations. Most individuals expressed neutral attitudes towards police enforcement (51%), followed by negative attitudes towards enforcement (31%). Overall, individuals were particularly concerned about impaired driving, followed by speeding and distracted driving. A large proportion of individuals expressed negative attitudes towards these behaviours, however, there were still a meaningful number of individuals who expressed positive attitudes towards these behaviours.
Vingilis et al., 2018	Three focus groups (of 2-hours duration) conducted with younger males aged between 18–30 years (mean age = 23), residing in Ottawa (n = 3), London (n = 8) and Toronto (n = 11). Participants were asked question regarding risky driving videos as well as shown two risky driving videos (one involving motorcyclists ghost riding and doing wheelies, and the other involving similar content but with the addition of police following the rider)	YouTube	Risky driving behaviour (e.g., stunts	Exposure to motor vehicle/driving content on YouTube ranged from “very, very rarely” to “almost all the time” Sharing YouTube videos was not common Participants preferred watching YouTube than Television Reasons for watching driving-related content on YouTube fell under the following categories: information/education, reviews of vehicles, driving skills, new technology and entertainment A large proportion of the sample expressed negative attitudes towards the risky driving behaviours (ghost riding and wheelies) in the YouTube videos, whilst some also expressed positive attitudes towards the behaviours (e.g., “the drivers were skilled” “that’s so cool, I wish I could do that. But at the same time, you’re like that’s so stupid. I would never do that”) Participants stated that feedback on social media (e.g., likes, comments etc) could promote further engagement in the behaviour in those who post such videos, whilst social media exposure could also influence the viewers’ behaviour (e.g., in younger groups or in those who are predisposed to engage in risk taking behaviours) Some admitted to engaging in these behaviours at some point (e.g., when they were younger) or had thought about engaging in the behaviour/s Some participants expressed that public service announcements targeting risky driving behaviours would not reach many individuals, and that such messages should be shared on social media rather than television

### Results within studies & synthesis of results

#### Driving-related content on YouTube

A total of 4 studies examined driving-related content on YouTube. Basch, Mouser [17] examined the nature of content in 100 distracted driving videos and found that videos originating from Television were more likely to contain phone use while driving material compared to consumer-based videos. Importantly, they were also more likely to mention that the behaviour was illegal. However, whilst a large proportion of the videos contained phone use while driving content, the nature of the content was not specified. For example, it was not clear as to how many videos encouraged versus discouraged distracted driving behaviour, and which types of videos were deemed more popular (e.g., had more views, comments etc).

Gjorgjievski, Sprague [18] found that whilst distracted driving videos (with 223 million views) on YouTube were mostly serious in nature (e.g., did not contain humorous material and/or highlighted the consequences of the behaviour), only 3.4% of videos reported data from peer-reviewed studies. In addition, Seeley, Wickens [19] found that comments linked to risky driving videos were both positive and negative, although the majority of the comments (84.7%) on the stunt videos were positive. Concerningly, the consequences of such behaviours were rarely mentioned.

#### Driving-related content on Twitter & other

Three studies reviewed the content of Tweets in attempting to understand trends in driver attitudes and behaviours. Stephens, Trawley [20] collected 80,923 tweets that were related to road rage and expressed aggression toward the perceived lack of skill of other drivers on the road. Interestingly, many of these tweets appeared to be posted while driving, suggesting that this trend of criticising other drivers on social media may be creating a dangerous distraction of its own.

Sujon and Dai [21] explored attitudes and beliefs towards road safety in Washington. Although a large proportion of individuals expressed negative attitudes towards impaired driving, speeding and distraction, there was still a notable proportion of individuals who expressed positive attitudes towards these behaviours. Such themes were also identified in Mooren, Grzebieta [22] study, which found a large body of content on social media chatrooms, blogs and webpages that expressed negative attitudes towards speed enforcement in Australia, labelling it as a “nanny state”.

#### Social media exposure & subsequent driving behaviour

Importantly, only one study examined the impact of social media exposure on driving behaviour. Vingilis, Yildirim-Yenier [23] utilised focus groups to explore reactions to risky driving YouTube videos among young males, finding that whilst the majority of the sample deemed the behaviours as foolish and dangerous, some individuals expressed mixed or even positive attitudes towards them (e.g., “the drivers were skilled” or “that’s so cool, I wish I could do that. But at the same time, you’re like that’s so stupid. I would never do that”). Further, there was an agreement among some participants that it could influence certain individuals (particularly those who were younger in age or were predisposed to engage in risky behaviour). Concerningly, a small number of participants mentioned that they had previously attempted the behaviour/s, although there was no direct examination between level and type of exposure upon self-reported behaviour.

## Discussion

Whilst there is emerging evidence to suggest that exposure to content on social media encouraging risky behaviours (such as substance use, disordered eating, or violent behaviour) increases the probability of engagement in the target behaviour [e.g., 4–6], the impact of social media content on road rule compliance has yet to be thoroughly examined. This study explored current peer-reviewed evidence concerning social media and how it relates to road safety and/or driving-related behaviour, aiming specifically to: (a) identify the nature of driving-related content on social media, and (b) to examine whether such content can affect attitudes and subsequent driving behaviour. A core finding was that only 8 studies were identified, which may be considered a significant disparity to the amount of driving-related content available on social media platforms. Whilst very few themes could be derived from this small sample, three key points warrant attention. First, whilst very limited research has explored the content on social media relating to driving behaviour, the majority of studies focus solely on YouTube and Twitter. This is in contrast to the array of social media platforms available to consumers, many of which are designed to facilitate social interactions and depict attitudes, perceptions and behaviours. Second, whilst the limited number of studies in this review uncovered content that promotes road safety or road rule compliance, a body of content exists that encourages risky driving or offending behaviour. Third, there is a paucity of applied research investigating the impact of driving-related content (on social media) upon self-reported attitudes, intentions and behaviours. That is, it remains unclear how (and to what extent and direction) social media exposure influences driving behaviours. This includes whether such subsequent behaviours are concealed or actively promoted on social media (creating further reinforcing mechanisms). In regard to the latter, no study to date has directly investigated whether such exposure creates measurable change in subsequent attitudinal and behavioural outputs, or merely reinforces behaviours for those who have a predisposition to offend.

Nevertheless, the current review revealed that half of the studies examined content on YouTube videos pertaining to distracted driving and risky driving behaviour (e.g., ghost riding and wheelies), whilst the remaining focused on Twitter content (tweets) and general social media chatrooms/blogs. In terms of YouTube, both negative and positive driving-related videos (i.e., videos that both encourage or discourage negative behaviours or road rule violations) were popular on this platform. Notably, the distracted driving content involved more videos that discouraged, as opposed to encouraged, the behaviour. This is most likely due to the search terms used in the studies, as it is acknowledged that YouTube videos that encourage distracted driving are likely to involve additional content, making the distracted driving component of the video a minor part of the content that cannot be captured by search terms. For example, YouTube vlog content may be unrelated to distracted driving, yet the vlogger may be engaging in this behaviour during the video which could be indirectly encouraging distracted driving; such content is unlikely to be identified by distracted driving related search terms. Meanwhile, only one study conducted a content analysis on YouTube videos that focused on risky driving behaviours (specifically, street racing, stunt driving and ghost riding), and it was found these videos are common, yet there is a mix of positive and negative attitudes towards the content [19].

Similar results were found on Twitter, with a mixture of content that encourages and discourages risky driving behaviour, with one study demonstrating that individuals even Tweet while driving [20]. Finally, one study found that a large body of social media blogs and webpages express negative attitudes towards speed enforcement, referring to Australia as the “nanny state” [22]. Overall, these findings suggests that whilst some efforts have been made to promote road safety and rule compliance on social media, there is a notable amount of content promoting or encouraging offending behaviour. Nonetheless, further research is warranted to examine the relative proportion of content that encourages, as opposed to discourages, risky driving behaviour online. Indeed, careful inspection of the data and methodologies indicates that all studies limited their searches to a single platform and a specific topic (e.g., distracted driving). As such, it is not possible to delineate the frequency and extent to which individuals are exposed to driving-related content on social media, relative to other topics of interest (e.g., health and fitness content, Covid-19). Further, the question of whether the content (and the popularity and saliency of the content) varies across platforms, is yet to be investigated. In order words, whether individuals are regularly exposed to driving-related content, and whether such content is equally or less popular than other topics (such as health and fitness) is not known.

On a similar note, and taking it a step further, it is completely unknown as to whether such content influences attitudes towards road safety (including perceptions of risk) and how this is operationalised into actual driving behaviours (e.g., rule compliance). Importantly, a lack of studies in this review precluded the ability to determine whether social media content influences driving behaviour. In fact, only one study attempted to address this question. Through focus groups, Vingilis [23] found that whilst young males generally expressed negative attitudes towards risky driving behaviour (labelling the behaviour as foolish or dangerous), they acknowledged that it could influence certain individuals. Interestingly, whilst Seeley, Wickens [19] did not examine this question directly, they found a number of “copycat videos where young men attempted to mimic the original ghost ride music video with their vehicles” (p. 291). This finding may have broader implications regarding theories of human learning, and highlights the need to consider the saliency and influence of social media on subsequent behaviour. Not surprisingly, this also has links to the perceived importance of adhering to road rules and/or the promotion of placement in high-risk situations that are not calibrated to an individual’s driving skill.

Due to a lack of research in this area, limited themes or conclusions can be drawn from this review regarding the relationship between social media exposure and driving behaviour. The fact that grey literature was not included in the analysis, may also be considered a limitation. Further, due to the nature of the studies included, a risk of bias assessment was not undertaken. Finally, we also acknowledge that a range of social media platforms exist, of which only 11 were listed as search terms in the present review. Nonetheless, this review highlights the need for research to keep pace with the rapidly changing nature and popularity of social media in the modern world (from both a theoretical and practical standpoint), and provides important avenues for future research investigating the impact of social media messaging on road rule compliance. First, there is a pressing need for research to investigate the nature and frequency of driving-related content on various social media platforms, and to determine the proportion of messages that pertain to road safety compared to other topics of interest. More specifically, research needs to examine driving-related content on additional social media platforms (such as Snapchat, Instagram, Facebook and TikTok), as well as identify the breadth of driving content across different platforms. This includes identifying whether such platforms primarily promote engagement in deliberate risky driving behaviours (e.g., impaired driving, tailgating) and/or creates secondary processes that are corrosive to road safety (e.g., changing attitudes and perceptions). Such knowledge will help inform future research attempting to directly quantify and elucidate the relationship between social media exposure and subsequent driving behaviour.

## Supporting information

S1 File Search strategy for all databases. (DOCX)

S1 Checklist PRISMA checklist. (DOC)
