# Supplementary material for: Does exposure to social media content influence attitudes towards, and engagement in, road rule violations? A systematic review
Source: PLoS One. 2022 Sep 28;17(9):e0275335. doi: 10.1371/journal.pone.0275335 (PMC9518855; doi:10.1371/journal.pone.0275335)
Supplement: S1 File — (DOCX) [file pone.0275335.s001.docx]

**Search Strategy for all Databases**

**Search terms**

(1) Social media, YouTube, Snapchat, Facebook, Instagram, Twitter, TikTok, Reddit, WhatsApp, Waze, maps, navigation application. (2) Traffic, road, drive*, driving**.** (3) Following distance, headway, tailgat*, dangerous, unsafe, distraction, texting, cellphone, phone, violation, offend*, hoon*, rules, drug, drink, impaired, seatbelt, speeding, speed limit.

**Search strategy**

SCOPUS (2,036)

TITLE-ABS ( {Social media} OR youtube OR snapchat OR facebook OR instagram OR twitter OR tiktok OR reddit OR whatsapp OR waze OR maps OR {Navigation Applications} ) AND TITLE-ABS ( traffic OR road OR drive* OR driving ) AND TITLE-ABS ( {Following distance} OR headway OR tailgat* OR dangerous OR unsafe OR distraction OR texting OR cellphone OR phone OR violation OR offend* OR hoon* OR rules OR drug OR drink OR impaired OR seatbelt OR speeding OR {speed limit} ) AND ( LIMIT-TO ( DOCTYPE , "ar" ) ) AND ( LIMIT-TO ( LANGUAGE , "English" ) )

PUBMED (356)

(({Social media} [Title/Abstract] OR youtube [Title/Abstract] OR snapchat [Title/Abstract] OR facebook [Title/Abstract] OR instagram [Title/Abstract] OR twitter [Title/Abstract] OR tiktok [Title/Abstract] OR reddit [Title/Abstract] OR whatsapp [Title/Abstract] OR waze [Title/Abstract] OR maps [Title/Abstract] OR {Navigation Applications}[Title/Abstract]) AND (traffic [Title/Abstract] OR road [Title/Abstract] OR drive* [Title/Abstract] OR driving[Title/Abstract])) AND ({Following distance} [Title/Abstract] OR headway [Title/Abstract] OR tailgat* [Title/Abstract] OR dangerous [Title/Abstract] OR unsafe [Title/Abstract] OR distraction [Title/Abstract] OR texting [Title/Abstract] OR cellphone [Title/Abstract] OR phone [Title/Abstract] OR violation [Title/Abstract] OR offend* [Title/Abstract] OR hoon* [Title/Abstract] OR rules [Title/Abstract] OR drug [Title/Abstract] OR drink [Title/Abstract] OR impaired [Title/Abstract] OR seatbelt [Title/Abstract] OR speeding [Title/Abstract] OR {speed limit}[Title/Abstract])

ProQuest (1,500)

(ab({Social media} OR youtube OR snapchat OR facebook OR instagram OR twitter OR tiktok OR reddit OR whatsapp OR waze OR maps OR {Navigation Applications} ) OR ti({Social media} OR youtube OR snapchat OR facebook OR instagram OR twitter OR tiktok OR reddit OR whatsapp OR waze OR maps OR {Navigation Applications} )) AND (ab(traffic OR road OR drive* OR driving ) OR ti(traffic OR road OR drive* OR driving )) AND (ab({Following distance} OR headway OR tailgat* OR dangerous OR unsafe OR distraction OR texting OR cellphone OR phone OR violation OR offend* OR hoon* OR rules OR drug OR drink OR impaired OR seatbelt OR speeding OR {speed limit}) OR ti({Following distance} OR headway OR tailgat* OR dangerous OR unsafe OR distraction OR texting OR cellphone OR phone OR violation OR offend* OR hoon* OR rules OR drug OR drink OR impaired OR seatbelt OR speeding OR {speed limit}))

Filters: *Scholarly journals / peer reviewed / English*

TRID (549)

({Social media} OR youtube OR snapchat OR facebook OR instagram OR twitter OR tiktok OR reddit OR whatsapp OR waze OR maps OR {Navigation Applications}) AND (traffic OR road OR drive* OR driving) AND ({Following distance} OR headway OR tailgat* OR dangerous OR unsafe OR distraction OR texting OR cellphone OR phone OR violation OR offend* OR hoon* OR rules OR drug OR drink OR impaired OR seatbelt OR speeding OR {speed limit} ) *Only articles and papers / English*
